# Evolutionary transition from single-cell to multi-cell organisms: the role of surface waters and their hydrodynamics

**DOI:** 10.1038/s41598-026-57306-7

**Published:** 2026-06-12

**Authors:** Vladimir Nikora, Hongwei Fang, Guojian He, Lei Huang

**Affiliations:** 1 School of Engineering, University of Aberdeen, Aberdeen, UK; 2 Department of Hydraulic Engineering, Tsinghua University, Beijing, China; 3 Department of Ocean Science and Engineering, Southern University of Science and Technology, Shenzhen, China

**Keywords:** Transition from single-cell to multi-cell organisms, Flow-microorganism interactions, Evolution of microorganisms, Environmental turbulence, Computational biology and bioinformatics, Ecology, Ecology, Evolution, Microbiology, Ocean sciences, Systems biology

## Abstract

The evolutionary transition from unicellular to multicellular organisms exhibited a sharp increase in metabolic rates, yet the physical conditions supporting this transition remain poorly understood. Here we propose that wide-spread hydrodynamic enhancement of nutrient delivery to the microorganisms in the Neoproterozoic Era constituted a necessary enabling condition for early multicellularity, complementing the effect of rising oxygen levels occurring at the same time. Along with conceptual arguments in support of this hypothesis, we define three testable hydrodynamic mechanisms (viscous-diffusive, viscous-convective, and inertial-convective) controlling nutrient uptake and largely applicable for both bed-attached and free-floating microorganisms. We also highlight a flow-bed interface in surface streams as potentially important hotbed for multicellularity breeding, complementing the mainstream perception on the defining role of early oceans. Overall, our results emphasize hydrodynamics as under-represented physical component in existing evolutionary frameworks and generate testable predictions linking organism size, flow conditions, and habitat type.

## Introduction

The initial living creatures on Earth emerged ca. 3.6-4.0 billion years ago as primitive unicellular organisms, with the next major step being the appearance of oxygen-producing cyanobacteria around a billion years later. A gradual rise of oxygen in aquatic systems and atmosphere provided favourable conditions to support more sophisticated multicellular organisms that required higher energy consumption compared to single-cellular organisms^1,2^. The data suggest that the burst of multicellular organisms on Earth, which led to the ‘Cambrian Explosion’ (ca. 550 − 500 Ma), occurred between ca. 1000 − 550 Ma (known as the Neoproterozoic Era) when oxygen levels were sharply increasing. This period, however, also coincided with times of a wide-spread formation of mountain systems and geochemical events that in the presence of surface freshwater from rains and melting glaciers helped form fast streams and rivers, carrying various dissolved minerals. Were these geophysical processes another critical factor required for the emergence of multicellular forms of life?

Although various scenarios for the mass emergence of multi-cellular organisms have been proposed in recent years^3,4^, the exact evolution routes from single cells to multicellularity remain largely unknown^5^. The precise mechanisms securing high metabolic rates needed for the initial multicellular microorganisms also await clarification. Some studies suggest a certain role of cooperative hydrodynamic effects associated with colonies of swimming and/or suspension-feeding unicellular organisms living in quiescent viscosity-dominated environments^6–8^. Indeed, the data and proposed models^6–9^ demonstrate advantages of colonial assemblages in terms of enhanced exchange of substances with surrounding environment. Bearing in mind that the rate of delivery of nutrients towards microorganisms is a measure of their overall metabolic rate^10–12^, one can argue that such colonies are likely to be a step forward towards multicellular organisms. Indeed, Short et al.^6^ demonstrated that once a colony of single cells is formed, their specific arrangements and the coordinated beating of surface-mounted flagella generate a diffusive boundary layer of a nutrient, thus enhancing its uptake. The authors showed that there is a ‘bottleneck’ size of a colony, above which the molecular diffusion starts to constrain metabolism. To overcome this constraint, a more efficient transport mechanism is needed to support larger organisms. Short et al.^6^ proposed that this mechanism relates to advection emerging as a result of synchronised motions of the flagella of external border cells of the colony. It should be noted that Short et al.’s^6^ paper was among first that explicitly demonstrated the need for advective transport of substances to support evolution to multicellularity in quiescent viscosity-dominated environments. But could cell-induced currents and/or cell mobility alone secure a sufficient increase in metabolic rates needed for multicellular organisms to evolve at a massive scale in a quiescent water environment? It seems unlikely as (1) the benefits of enhanced delivery of nutrients would be limited by the need to spend additional energy to create and maintain diffusive boundary layers around organisms, and (2) the external background concentration of nutrients would be another limiting factor as their replenishment in quiescent water environments would be constrained, again, by molecular diffusion. The enrichment of atmosphere and quiescent aquatic systems with oxygen may not help either, although it is undoubtedly one of the key conditions for the mass appearance of multicellular forms of life. But what was another necessary condition for this, a condition that may help explain the bursting development of multicellular organisms within a geologically short period of time around ca. 1000 − 550 Ma?

Here we propose a hypothesis that this another *necessary condition* was a need for a sharp increase in flow velocities and turbulence levels in aquatic systems. Together with boosted concentrations of oxygen and other nutrients, hydrodynamically enhanced delivery of substances towards organisms and removal of wastes could be a key mechanism securing metabolic rates, which are much higher than those constrained by molecular diffusion in quiescent environments. The increased flow turbulence could also modulate other drivers such as predation or symbiosis. As already mentioned, the organism-induced currents or cell mobility may enhance metabolic rates of microorganisms in slow waters, but they are not adequate for securing sufficiently high metabolic rates for wide-ranging multicellular organisms. Indeed, the so called Peclet number (a ratio of convective transport to molecular diffusion) for unicellular organisms is relatively small while the Reynolds number (a ratio of inertial forces to viscos forces) of quiescent environment is too low for providing required level of mass transfer compared to action of turbulence^1,9–15^.

The effects of flow hydrodynamics on the microorganisms are multifaceted and may also differ depending on their habitats and specific lifestyles^12,14–16^, e.g., free-floating microorganisms react to turbulent motions differently from sessile microorganisms growing on submerged surfaces. Nevertheless, wide-spread enhancement of mean flow velocities and turbulence levels in the Neoproterozoic surface waters affected the living conditions of them both, widely opening a physical ‘metabolic window’ for the emergence of multicellular microorganisms. Even though it is very likely that there have been occasional turbulent spots in surface waters in the preceding period, only in the Neoproterozoic Era they became lasting and widely spread. Below we first outline a conceptual framework followed by potential scenarios of hydrodynamic effects on the nutrient delivery to the microorganisms, concluding with suggestions for future studies and empirical tests. Our main focus is on the habitats of streams, rivers, and sheet-like surface flows abundant in the Neoproterozoic Era. Although it is common to assume that multicellular organisms mainly evolved in early oceans, the potentially significant role of surface freshwater flows should not be neglected, and this article will hopefully attract attention to this potential evolutionary route.

## Results

### Conceptual framework

**Fig. 1:**
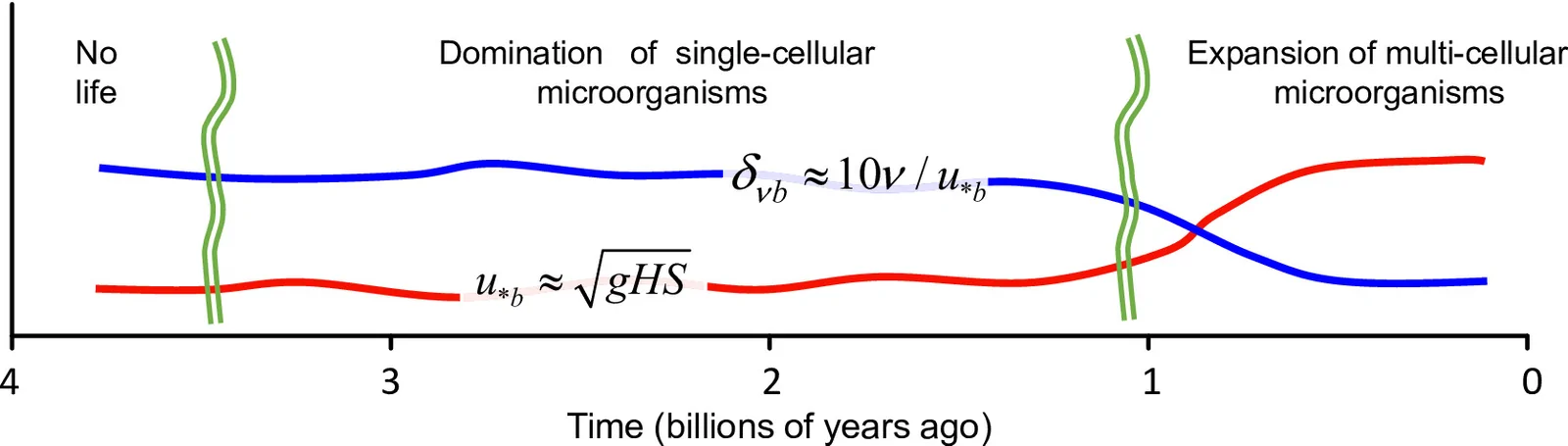
Sketch showing the hypothetical time evolution of viscous sublayer thickness $\:{\delta\:}_{\nu\:b}\approx\:10\nu\:/{u}_{*b}$ and shear velocity $\:{u}_{*b}\approx\:\sqrt{gHS}$,  where *ν* is fluid kinematic viscosity, *g* is gravity acceleration, *H* is flow depth, and *S* is bed (terrain) slope. Subscript *b* means that the shear velocity and thickness of viscous sublayer relate to a flow over a stream bed. The sharp changes in $\:{\delta\:}_{\nu\:b}\approx\:10\nu\:/{u}_{*b}$ and $\:{u}_{*b}\approx\:\sqrt{gHS}$ around ca. 800 M years ago reflect conditions of the Neoproterozoic mountain growth.

Considering freshwater streams on the Earth surface as a major seedbed for breeding multicellular organisms at the water-solid interfaces^16^, we observe that flow velocity and turbulence parameters in such high-Reynolds number streams are closely scaled by so-called ‘shear velocity’ $\:{u}_{*b}$, which is proportional to the square root of the product of bed slope *S* and flow depth *H* (i.e.,  $\:{u}_{*b}\approx(gHS{)}^{1/2}$, where *g* is gravity acceleration^17,18^. This suggests that mean velocities and turbulence levels in surface streams would unavoidably rise in response to increasing slopes *S* of the terrain and/or depths *H* of surface water flows (Fig. 1).

**Fig. 2:**
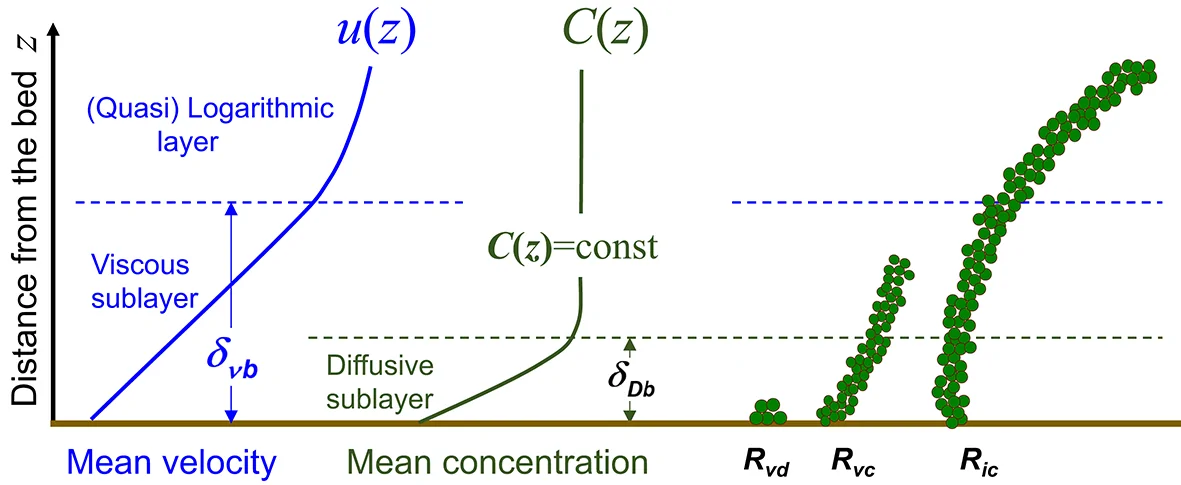
Sketch showing distribution of mean (time-averaged) flow velocity and nutrient concentration along direction orthogonal to the solid surface absorbing nutrients. This surface can be a bed surface covered by microorganisms or surfaces of individual microorganisms themselves. Three proposed nutrient uptake regimes are also shown: (1) *viscous-diffusive* regime (*R*_*vd*_) when $\:L<{\delta\:}_{Db}$; (2) *viscous-convective* regime (*R*_*vc*_) when $\:{\delta\:}_{Db}<L<{\delta\:}_{\nu\:b}$; and (3) the *inertial-convective* regime (*R*_*ic*_) when $\:L>{\delta\:}_{\nu\:b}$, where *L* is a representative size of a microorganism.

At times of mass expansion of multicellular organisms, both these quantities experienced enhancements due to accelerated mountain growth^19^, increased rains^20^, and ice melting^21^. Interestingly, there is evidence that massive mountain growth could itself be triggered by unicellular organisms (cyanobacteria) that deposited on the ocean bed, lubricating mountain building^22^. In turn, this process could lead to enhanced turbulence in water streams and thus to accelerated emergence of multicellular organisms. For the sake of this consideration, a turbulent stream on the Earth surface could be subdivided into specific (sub)layers defined by the dominant transport mechanisms. In relation to flow velocity, there are: (1) a thin layer ‘attached’ to the solid surface, where viscous momentum transport is dominant and where flow velocity increases quasi-linearly from zero at the bed; this layer is known as a *viscous sublayer* with thickness $\:{\delta\:}_{\nu\:}$ inversely proportional to the shear velocity $\:{\delta\:}_{\nu\:}\approx\:10\nu\:/{u}_{*}$^17,18^ where *ν* is water kinematic viscosity; and (2) a layer above where momentum transport mechanisms are driven by turbulence and where velocity distribution is quasi-logarithmic (known as an *outer layer*), Fig. 2. These two distinct layers of momentum transport towards the streambed are superimposed with a similar two-layer structure for transport of substances: (1) a near-bed layer where molecular diffusion of substances is dominant and where nutrient concentration changes quasi-linearly from the concentration at the bed; this layer is known as a *diffusive sublayer* of thickness $\:{\delta\:}_{D}$ which for the relevant nutrients is approximately an order of magnitude thinner than a viscous sublayer, i.e., $\:{\delta\:}_{D}=S{c}^{-1/3}{\delta\:}_{\nu\:}\approx\:0.1{\delta\:}_{\nu\:}$ where $\:Sc=\nu\:/D$ is Schmidt number with $\:Sc\approx\:500-2000$ for compounds of oxygen, carbon, hydrogen, and nitrogen^23^; and (2) a layer above where mixing and transport of substances is driven by turbulence and thus the concentration is approximately constant due to intense turbulent mixing, Fig. 2. The outlined specific layers in velocity and concentration distributions are smoothly linked by thin interfacial regions which, as a first approximation, can be neglected in our consideration. A similar conceptual picture is largely valid for any solid surface contacting with turbulent water flow, including organism surfaces. In case of streambed-related sublayers, we add a subscript *b* to the relevant symbols, whereas for the sublayers associated with the microorganisms themselves, we add a subscript *o*.

**Fig. 3:**
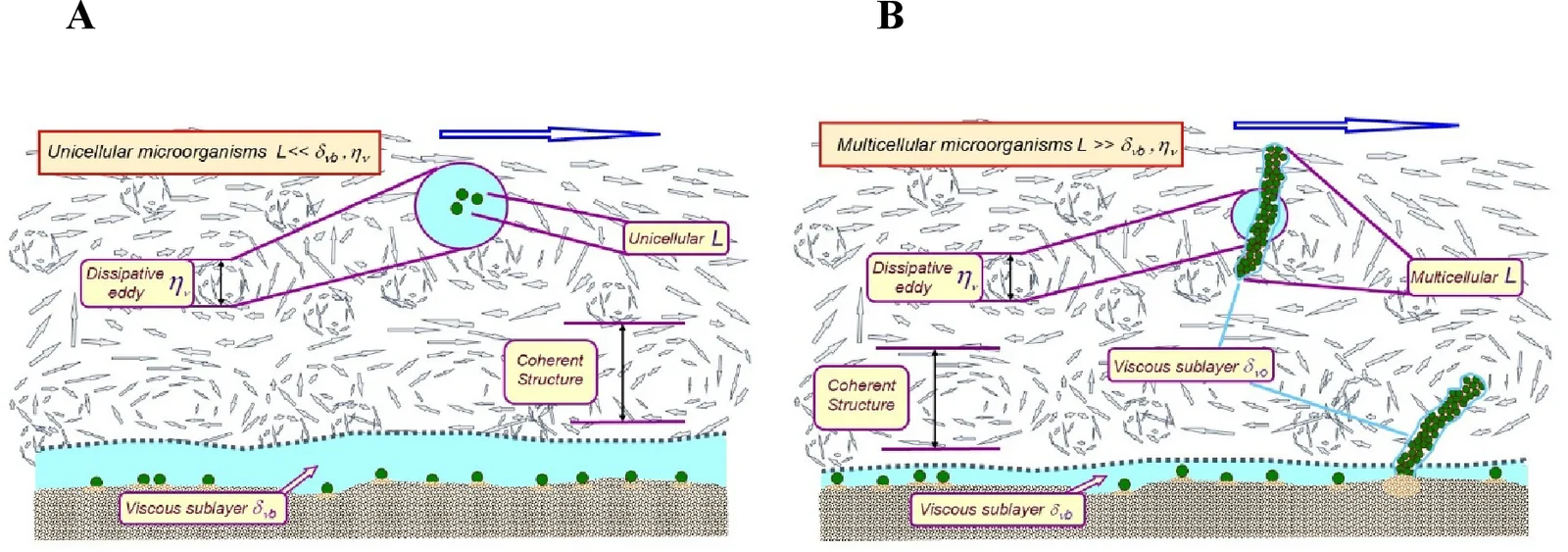
Interrelations of microorganisms with viscous-dominated flow regions. (**A**) Unicellular microorganisms are deeply embedded into bed-attached viscous sublayer of thickness $\:{\delta\:}_{\nu\:b}$ and Kolmogorov’s dissipative eddies of scale $\:{\eta\:}_{\nu\:}$, thus being highly dependent on slow molecular diffusion. (**B**) Multicellular microorganisms with dimensions *L* well exceeding $\:{\delta\:}_{Db}$ and $\:{\eta\:}_{D}$, exposed to higher velocities and thus surrounded by thinner viscous sublayers, allowing larger fluxes of oxygen and other nutrients towards the cell surfaces. The multicellular microorganisms in **B** are shown as aggregations of cells, but similar considerations also apply for microorganisms evolving from one cell via a ‘clonal’ trajectory to multicellularity. The diffusion-dominated regions embedded into viscos-dominated regions as outlined in Fig. 2 are not shown to prevent cluttering the sketches.

With enhancement of mean flow velocities and turbulence levels, the substances (e.g., oxygen and other relevant nutrients) within the outer layer are quickly mixed by turbulence providing their fast delivery towards the upper boundary of the diffusive sublayer at the streambed covered by single-cell organisms (Figs. 2 and 3). However, the transport within the diffusive sublayer is several orders of magnitude slower, becoming a critical bottleneck in delivery of substances required for microorganisms living on the bed surface. The diffusive flux towards the bed and organism surfaces can be described by Fick’s law of molecular diffusion stating that it is equal to the product of the molecular diffusivity and the gradient of substance concentration within the diffusive sublayer^13,23^. Thus, with all other factors equal, the transport rate through the diffusive sublayer can be sharply increased if the thicknesses of the viscous and diffusive sublayers are noticeably reduced (thus increasing the concentration gradient), as will be explicitly shown in the next section (see also Figs. 2 and 3). This reduction can be achieved by increasing the shear velocity, which can be realised via increasing the streambed slope *S* and/or water depth *H*, as $\:{\delta\:}_{Db}\approx\:0.1{\delta\:}_{\nu\:b}\approx\:\nu\:/{u}_{*b}\approx\:\nu\:/(gHS{)}^{1/2}$. Once the thickness of the diffusive sublayer $\:{\delta\:}_{Db}$ (associated with the streambed) becomes smaller than the organism size (Figs. 2 and 3), the thickness of the diffusive sublayer $\:{\delta\:}_{Do}$ (surrounding an organism’s surface, Fig. 3b) will become the main barrier for delivery of substances to the organism. As the organism-related diffusive sublayer is much thinner than that of the streambed (i.e., $\:{\delta\:}_{Do}$<<$\:{\delta\:}_{Db}$), the flux of substances is enhanced, highlighting a benefit of being taller than the thickness of the streambed-related diffusive sublayer. Since the thicknesses of the viscous and diffusive sublayers are inversely proportional to the shear velocity $\:{u}_{*b}$, the nutrient uptake rate should be dependent on $\:{u}_{*b}$ (i.e., on the terrain slope and flow depth). This dependence is strongly supported by the measurements of nitrogen and phosphorus uptake rates by streambed algae^24^. The microorganism-induced currents and/or mobility can further boost the delivery of nutrients at the organism scale^6^ but their overall contribution is likely to be minor compared to the enhanced hydrodynamics of the habitat.

The described conceptual picture could be further expanded to take into consideration the bed sediments, as voids and cavities at flow-sediment interfaces could provide additional advantages for the aggregation (flocculation) of individual cells and the emergence of multicellular organisms^25^. Although the above considerations relate to the bed-attached organisms, physically similar effects of turbulence also apply in relation to free-floating microorganisms (Fig. 3). To support the above conjectures, below we briefly outline potential scenarios at streambeds (bed-attached cells) and in the flow above (floating cells), which may explain enhancements of metabolic rates of microorganisms.

### Potential scenarios for hydrodynamic effects on the nutrient uptake by surface-attached microorganisms

Based on the conceptual picture outlined in the previous section and in Figs. 2 and 3, here we introduce three regimes of the nutrient uptake by the microorganisms: (1) *viscous-diffusive* regime ($\:{R}_{vd}$) for $\:L<{\delta\:}_{Db}$; (2) *viscous-convective* regime ($\:{R}_{vc}$) when $\:{\delta\:}_{Db}<L<{\delta\:}_{\nu\:b}$; and (3) *inertial-convective* regime ($\:{R}_{ic}$) when $\:L>{\delta\:}_{\nu\:b}$, where *L* is a vertical size of a microorganism in this case. The rate of delivery of nutrients to an organism surface can be quantified as $\:R={\int\:}_{A}D(dC/d{x}_{j}){n}_{j}da$, where *D* is molecular diffusion coefficient, *C* is nutrient concentration, $\:dC/d{x}_{j}$ is spatial gradient of nutrient concentration, *n*_*j*_ is unit inward vector, and *A* is an organism surface area. The terms *viscous-diffusive*, *viscous-convective*, and *inertial-convective* are borrowed from the spectral theory for passive scalars in turbulent flows^26,27^, and we use similar terms here and in the next section on floating microorganisms as they appropriately reflect the essence of relevant transport mechanisms.

For the *viscous-diffusive* regime ($\:{R}_{vd}$, $\:L<{\delta\:}_{Db}$) we can approximate the nutrient uptake rate as:

1 $$\:{R}_{vd}={\int\:}_{A}D\frac{dC}{d{x}_{j}}{n}_{j}da\propto\:AD\frac{{\Delta\:}{C}_{{\delta\:}_{Db}}}{{\delta\:}_{Db}}\propto\:ADS{c}^{1/3}\frac{{\Delta\:}{C}_{{\delta\:}_{Db}}}{{\delta\:}_{\nu\:b}}\propto\:ADS{c}^{1/3}{\Delta\:}{C}_{{\delta\:}_{Db}}\frac{{u}_{*b}}{\nu\:}\propto\:{\Delta\:}{C}_{{\delta\:}_{Db}}{L}^{\beta\:}\frac{{u}_{*b}}{\nu\:}$$

where $\:{u}_{*b}$ is shear velocity related to a viscous sublayer at the bed, $\:{\delta\:}_{Db}=S{c}^{-1/3}{\delta\:}_{\nu\:b}$, $\:Sc=\nu\:/D$ is the Schmidt number, and where we expressed an organism surface area as $\:A\sim{L}^{\beta\:}$. The exponent $\:\beta\:$ ranges, depending on the organism shape, from nearly 1.0 for filamentous shapes to 2.0 for three-dimensional shapes^28^, or even higher for the case of fractal surfaces. In Eq. (1) we used an approximation that the thickness of the organism-related diffusive sublayer is equal to that of the diffusive sublayer at the bed surface (i.e., $\:{\delta\:}_{Do}\approx\:{\delta\:}_{Db}$) as $\:L<{\delta\:}_{Db}$. Similarly, for the *viscous-convective* regime ($\:{R}_{vc}$, $\:{\delta\:}_{Db}<L<{\delta\:}_{\nu\:b}$) the uptake rate can be expressed as:

2 $$\:{R}_{vc}\propto\:ADS{c}^{1/3}\frac{{\Delta\:}{C}_{{\delta\:}_{Do}}}{{\delta\:}_{\nu\:o}}\propto\:ADS{c}^{1/3}{\Delta\:}{C}_{{\delta\:}_{Do}}\frac{{u}_{*o}}{\nu\:}\propto\:ADS{c}^{1/3}{\Delta\:}{C}_{{\delta\:}_{Do}}\frac{{u}_{*b}^{2}L}{{\nu\:}^{2}}\propto\:{\Delta\:}{C}_{{\delta\:}_{Do}}{L}^{\beta\:+1}{\left(\frac{{u}_{*b}}{\nu\:}\right)}^{2}$$

where $\:{u}_{*o}$ is the shear velocity related to a viscous sublayer at the organism surface, which can be approximated using a local flow velocity $\:\overline{u}$ at the organism level (i.e., $\:{u}_{*o}\approx\:0.10{\overline{u}}_{L}$^29,30^, with an overbar defining time-averaging. Since in this regime the whole organism is embedded into the viscous sublayer at the bed, the local mean velocity is linearly increasing with distance from the bed (i.e., $\:{\overline{u}}_{L}={u}_{*b}^{2}L/\nu\:$^17,18^ and thus $\:{u}_{*o}\approx\:0.10({u}_{*b}^{2}L/\nu\:)$. Considering the third, *inertial-convective* regime ($\:{R}_{ic}$, $\:L>{\delta\:}_{\nu\:b}$), with an organism well protruding into a turbulent region above the viscous sublayer at the bed, and employing similar scaling arguments as above, we obtain:

$$\:{R}_{ic}\propto\:ADS{c}^{1/3}{\Delta\:}{C}_{{\delta\:}_{Do}}\frac{{u}_{*o}}{\nu\:}\propto\:ADS{c}^{1/3}{\Delta\:}{C}_{{\delta\:}_{Do}}\frac{1}{\nu\:}\left(\frac{{u}_{*b}}{\kappa\:}{ln}(\frac{{u}_{*b}L}{\nu\:})+{u}_{*b}B\right)\propto\:$$

3 $$\:{\Delta\:}{C}_{{\delta\:}_{Do}}{L}^{\beta\:}\frac{{u}_{*b}}{\kappa\:\nu\:}\left({ln}(\frac{{u}_{*b}L}{\nu\:})+\kappa\:B\right)$$

where we approximated vertical distribution of local mean flow velocity $\:\overline{u}$ with a semi-logarithmic equation $\:\overline{u}/{u}_{*b}=(1/\kappa\:{ln}({u}_{*b}z/\nu\:)+B$ with constants $\:\kappa\:\approx\:0.40$ and $\:B\approx\:5.0$^17,18^. Expression (3) and constants $\:\kappa\:$ and * B* relate to the so-called hydraulically-smooth-bed flow (with roughness elements fully submerged within the viscous sublayer); they may slightly change if the bed is hydraulically-rough^17,18^ but the message of Eq. 3 will remain.

Comparing Eq. (1) to (3) under an assumption that all factors are the same except hydrodynamics (i.e., bed-related shear velocity), one can observe that the nutrient uptake rates in the *viscous-convective* (Eq. 2, $\:{\delta\:}_{Db}<L<{\delta\:}_{\nu\:b}$) and *inertial-convective* (Eq. 3, $\:L>{\delta\:}_{\nu\:b}$) regimes well exceed that for the *viscous-diffusiv*e regime (Eq. 1, $\:L<{\delta\:}_{Db}$). The highest rate of change in the uptake increase occurs in the viscous-convective regime $\:{\delta\:}_{Db}<L<{\delta\:}_{\nu\:b}$, which seems to be the most plausible first step in the transition to multicellularity. Considering hydrodynamic conditions of modern low-slope lowland and high-slope mountain rivers^17^ as proxies for the ‘pre-multicellular’ and ‘multicellular’ conditions, respectively, we find that the shear velocity would be in the range of 1 cm/s to 3 cm/s for the former and 10 cm/s – 25 cm/s for the latter. These values convert into the values of the thicknesses of the viscous and diffusive sublayers as $\:{\delta\:}_{\nu\:b}=10\nu\:/{u}_{*b}\approx\:$300 –1000 μm and $\:{\delta\:}_{Db}=S{c}^{-1/3}{\delta\:}_{\nu\:b}\approx\:$30 –100 μm for the ‘pre-multicellular’ conditions, sharply reducing with the mountains growth to $\:{\delta\:}_{\nu\:b}\approx\:$40 –100 μm and $\:{\delta\:}_{Db}\approx\:$4 –10 μm. These approximate ranges agree well with dimensions of the relevant microorganisms, allowing to claim that even keeping all other factors the same, the enhanced flow conditions would grant a sharp increase in metabolic rates and thus secure the transition from single-cellular to multicellular organisms. Equation (1) to (3) also show that the hydrodynamic effects could be further enhanced with increase in the size of microorganisms who could change from one uptake regime to another during their development even in unchanged hydrodynamic conditions (Figs. 2 and 3). An independent data support for the above considerations can be found in Larned et al.^24^ who demonstrated a strong dependence of the uptake rate of nitrate and phosphorus by bed algae on the flow shear velocity.

### Potential scenarios for hydrodynamic effects on the nutrient uptake by free-floating microorganisms

It is unlikely that free-floating multicellular microorganisms could emerge in surface streams, as the travel time from the source to the ocean would be too short. However, the bed-attached microorganisms could be separated from the bed under the action of drag forces imposed by the flow, to become free-floating either attached to re-suspended fine sediment particles or independently. Due to turbulent mixing, these microorganisms could encounter and assemble into clusters and then either travel to the ocean or settle on the streambed and continue their further development being attached to the bed surface. Based on the available information and models of bio-sediments^25^ and microbial clustering^11,12^, we believe that this scenario of two-mode development (surface-attached and free-floating) is quite plausible.

A consideration, similar to the case of bed-attached microorganisms at sufficiently high Reynolds and Peclet numbers, could also apply for the case of freely moving microorganisms in the outer layer of surface streams (Fig. 3). The role of the viscous sublayer in this case is played by so-called Kolmogorov’s dissipative eddies, which are the smallest eddies as viscous forces at their scale dominate over inertia. In surface water flows, the size of these eddies is controlled by the shear velocity $\:{u}_{*b}$ and, in addition, by the distance *z* from the bed, $\:{\eta\:}_{\nu\:}=({\nu\:}^{3}/\epsilon\:{)}^{1/4}\approx\:({\nu\:}^{3}\kappa\:z/{u}_{*b}^{3}{)}^{1/4}\approx\:0.18{\delta\:}_{\nu\:b}^{3/4}(\kappa\:z{)}^{1/4}$, where $\:{\eta\:}_{\nu\:}$ is Kolmogorov’s dissipative scale $\:{\lambda\:}_{\nu\:}$ (~ 10 times smaller than the real size of a dissipative eddy, i.e., $\:{\lambda\:}_{\nu\:}=\alpha\:{\eta\:}_{\nu\:}$, where$\:\alpha\:\approx\:10$^26,27^, and $\:\epsilon\:$ is the dissipation of the turbulent kinetic energy that can be approximated in our case as $\:\epsilon\:\approx\:{u}_{*b}^{3}/\left(\kappa\:z\right)$^17,18^. Within a dissipative eddy one can distinguish a region dominated by the molecular diffusion. This region is similar, to a certain degree, to the diffusive sublayer imbedded into the viscous sublayer at the bed. Its physical size $\:{\lambda\:}_{D}$ can be quantified using Batchelor’s scale $\:{\eta\:}_{D}=(\nu\:{D}^{2}/\epsilon\:)$ as $\:{\lambda\:}_{D}=\alpha\:{\eta\:}_{D}$ where the constant can be assumed to be $\:\alpha\:\approx\:10$, as in relation $\:{\lambda\:}_{\nu\:}=\alpha\:{\eta\:}_{\nu\:}$ for Kolmogorov’s dissipative eddy. Similar to the interrelation $\:{\delta\:}_{D}=S{c}^{-1/3}{\delta\:}_{\nu\:}$ between the thicknesses of diffusive and viscous sublayers, one can deduce a relationship linking Kolmogorov’s ($\:{\eta\:}_{\nu\:}$) and Batchelor’s ($\:{\eta\:}_{D}$) scales as $\:{\eta\:}_{D}=S{c}^{-1/2}{\eta\:}_{\nu\:}$ or, equivalently, $\:{\lambda\:}_{D}=S{c}^{-1/2}{\lambda\:}_{\nu\:}$, $\:Sc=\nu\:/D$^11,26,27^. Thus, for a surface water flow we can obtain the interrelations $\:{\eta\:}_{\nu\:}=(\nu\:/{u}_{*b}{)}^{3/4}(\kappa\:z{)}^{1/4}\approx\:0.14{\delta\:}_{\nu\:b}^{3/4}{z}^{1/4}$ and $\:{\eta\:}_{D}=S{c}^{-1/2}(\nu\:/{u}_{*b}{)}^{3/4}(\kappa\:z{)}^{1/4}\propto\:0.025{\delta\:}_{Db}^{3/4}{z}^{1/4}$ explicitly showing that the characteristic scales $\:{\eta\:}_{\nu\:}$ and $\:{\eta\:}_{D}$ of dissipative eddies, defined by fluid kinematic viscosity and molecular diffusivity, are essentially inversely proportional to the shear velocity, similar to the thicknesses of viscous and diffusive sublayers formed at a solid surface.

Following scaling considerations as in the previous section, we can consider approximations for the three uptake regimes: (1) *viscous-diffusive* regime ($\:{R}_{vd}^{{\prime\:}}$) for $\:L<{\lambda\:}_{D}=\alpha\:{\eta\:}_{D}$; (2) *viscous-convective* regime ($\:{R}_{vc}^{{\prime\:}}$) when $\:{\lambda\:}_{D}=\alpha\:{\eta\:}_{D}<L<{\lambda\:}_{\nu\:}=\alpha\:{\eta\:}_{\nu\:}$; and (3) the *inertial-convective* regime ($\:{R}_{ic}^{{\prime\:}}$) when $\:L>{\lambda\:}_{\nu\:}=\alpha\:{\eta\:}_{\nu\:}$, where *L* is a representative size of a free-floating microorganism, and $\:\alpha\:\approx\:10$ as data suggest^26,27^. To help approximate the relevant uptake rates, we can use so-called velocity structure functions^26,27^ that characterise representative velocities acting on microorganisms within corresponding scale ranges (i.e., $\:L<\alpha\:{\eta\:}_{D}$, $\:\alpha\:{\eta\:}_{D}<L<\alpha\:{\eta\:}_{\nu\:}$, or $\:L>\alpha\:{\eta\:}_{\nu\:}$).

For the *viscous-diffusive* regime ($\:{R}_{vd}^{{\prime\:}}$, $\:L<{\lambda\:}_{D}=\alpha\:{\eta\:}_{D}$) we can assess the rate of the nutrient uptake by an organism as:

4 $$\:{R}_{vd}^{{\prime\:}}={\int\:}_{A}D\frac{dC}{d{x}_{j}}{n}_{j}da\propto\:AD\frac{{\Delta\:}{C}_{{\delta\:}_{Do}}}{{\delta\:}_{Do}}\infty\:\:ADS{c}^{1/2}\frac{{\Delta\:}{C}_{{\delta\:}_{Do}}}{{\lambda\:}_{\nu\:}}\propto\:{\Delta\:}{C}_{{\delta\:}_{Do}}{L}^{\beta\:}{\left(\frac{\epsilon\:}{{\nu\:}^{3}}\right)}^{1/4}\propto\:\text{}{\Delta\:}{C}_{{\delta\:}_{Do}}{L}^{\beta\:}{\left(\frac{{u}_{*b}^{3}}{{\nu\:}^{3}\kappa\:z}\right)}^{1/4}$$

where all variables are defined in the preceding text. In Eq. (4), we used an approximation that the thickness $\:{{\delta\:}_{D}}_{o}$ of the diffusive region surrounding a floating microorganism is equal to $\:{\lambda\:}_{D}$ (i.e., $\:{\delta\:}_{Do}\approx\:{\lambda\:}_{D}$), and that $\:{\lambda\:}_{D}=S{c}^{-1/2}{\lambda\:}_{\nu\:}$. Similarly, for the *viscous-convective* regime ($\:{R}_{vc}^{{\prime\:}}$, $\:{\lambda\:}_{D}=\alpha\:{\eta\:}_{D}<L<{\lambda\:}_{\nu\:}=\alpha\:{\eta\:}_{\nu\:}$) the uptake rate can be parameterised as below:

5 $$\:{R}_{vc}^{{\prime\:}}\propto\:AD\frac{{\Delta\:}{C}_{{\delta\:}_{Do}}}{{\delta\:}_{Do}}\infty\:ADS{c}^{1/3}\frac{{\Delta\:}{C}_{{\delta\:}_{Do}}}{{\delta\:}_{\nu\:o}}\propto\:ADS{c}^{1/3}{\Delta\:}{C}_{{\delta\:}_{Do}}\left(\frac{{u}_{*o}}{\nu\:}\right)\infty\:\text{}{\Delta\:}{C}_{{\delta\:}_{Do}}{L}^{\beta\:+1}{\left(\frac{\epsilon\:}{{\nu\:}^{3}}\right)}^{1/2}\infty\:{\Delta\:}{C}_{{\delta\:}_{Do}}{L}^{\beta\:+1}{\left(\frac{{u}_{*b}^{3}}{\nu\:^{3}\kappa\:z}\right)}^{1/2}$$

where $\:{u}_{*o}$ is the shear velocity related to a viscous sublayer at the surface of a floating organism, which can be approximated using a relevant local flow velocity $\:{u}_{L}$ at the organism scale so we can use a relationship $\:{u}_{*o}\approx\:0.10{u}_{L}$^28,29^, already employed in Eq. (2). Since we deal here with a free-floating organism, we should consider a representative velocity difference across an organism body to represent $\:{u}_{L}$. For this, we can use a second-order velocity structure function $\:\overline{{\Delta\:}{u}^{2}}\left(r\right)=\overline{{\left[u(x+r)-u\left(x\right)\right]}^{2}}$ where *r* is a separation distance between points where velocities are sampled, and an overbar defines ensemble averaging. For the viscous-convective regime, the relevant expression is $\:\overline{{\Delta\:}{u}^{2}}\left(r\right)\sim(\epsilon\:/\nu\:){r}^{2}$ for the subrange of dissipative eddies allowing specification of $\:{u}_{L}$ as $\:{u}_{L}\sim(\epsilon\:/\nu\:{)}^{1/2}L$, that we employed in Eq. (5)^17,26,27^. With further increase in the organism size (or decrease of $\:{\lambda\:}_{\nu\:}$ keeping the same size), the organism protrudes into a turbulent region outside Kolmogorov’s dissipative eddy marking a transition to the *inertial-convective* regime ($\:{R}_{ic}$, $\:L>{\delta\:}_{\nu\:b}$). Employing similar scaling arguments as for Eq. (5), we obtain:

$$\:{R}_{ic}^{{\prime\:}}\propto\:ADS{c}^{1/3}{\Delta\:}{C}_{{\delta\:}_{Do}}\frac{{u}_{*o}}{\nu\:}\propto\:S{c}^{1/3}{\Delta\:}{C}_{{\delta\:}_{Do}}{L}^{\beta\:}\frac{(\epsilon\:L{)}^{1/3}}{\nu\:}\propto\:{\Delta\:}{C}_{{\delta\:}_{Do}}{L}^{\beta\:+1/3}{\left(\frac{\epsilon\:}{{\nu\:}^{3}}\right)}^{1/3}\propto\:$$

6 $$\:\:{\Delta\:}{C}_{{\delta\:}_{Do}}{L}^{\beta\:+1/3}{\left(\frac{{u}_{*b}^{3}}{{\nu\:}^{3}\kappa\:z}\right)}^{1/3}$$

where the relative velocity at the organism scale is parameterised using an expression of the second-order structure function for the inertial subrange of turbulent scales, known as Kolmogorov’s 2/3rds law, i.e., in $\:\overline{{\Delta\:}{u}^{2}}\left(r\right)\sim{\epsilon\:}^{2/3}{r}^{2/3}$, and therefore $\:{u}_{*o}\approx\:0.10{u}_{L}\sim(\epsilon\:L{)}^{1/3}$.

As in the case of Eq. (1) to (3), the scaling relations (4) to (6) suggest that the uptake rates within the selected regimes, although somewhat different from those for the bed-attached organisms, increase with the shear velocity $\:{u}_{*b}\approx\:\sqrt{gHS}$, energy dissipation $\:\epsilon\:$, and organism size *L*. Also, Eq. (4) to (6) exhibit a trend for the uptake rates to decrease away from the bed. Similar to the bed-attached microorganisms, our scaling analysis predicts that the highest rate of change in the uptake increase occurs in the viscous-convective regime $\:\alpha\:{\eta\:}_{D}<L<\alpha\:{\eta\:}_{\nu\:}$, which is likely was the most plausible first step in the transition to multicellularity from hydrodynamics perspective.

If we again consider modern shallow lowland and mountain rivers^17^ as proxies for the ‘pre-multicellular’ and ‘multicellular’ flow conditions, we can deduce the dimensions $\:{\lambda\:}_{\nu\:}$ and $\:{\lambda\:}_{D}$ of the viscous and diffusive regions. For the ‘pre-multicellular’ conditions near the bed ($\:z\approx\:100$ mm) we obtain $\:{\lambda\:}_{\nu\:z}\approx\:$2000 –4400 μm and $\:{\lambda\:}_{D}\approx\:$60 –140 μm. The corresponding values sharply reduce with the mountains’ growth to $\:{\lambda\:}_{\nu\:}\approx\:$400 –800 μm and $\:{\lambda\:}_{D}\approx\:$10 –25 μm. These approximate estimates agree well with dimensions of relevant microorganisms and suggest that even relatively modest enhancement of hydrodynamic conditions should lead to the sharply increased rates of delivery of nutrients towards free-floating microorganisms. An independent qualitative support to our conjecture in this section can be found in Cornwallis et al.^4^ who reported their laboratory experiments on microorganism evolution and highlighted the role of turbulence. Unfortunately, the specific turbulence characteristics have not been quantified in their insightful experiments. On the other side, the proposed scaling relations agree well, at least qualitatively and conceptually, with findings from studies of plankton^10–12,14^ and freshwater bacteria^15^.

## Discussion

The proposed uptake scenarios highlight a potential role of flow hydrodynamics at the streambed and in the outer flow region in enhancing metabolic rates of the microorganisms in the Neoproterozoic Era. The outlined conceptual framework and proposed scenarios point out to a flow-bed interface as an important hotbed for multicellularity breeding, while the outer flow region could play at least a supporting function. The role of the viscous sublayer in this case is played by Kolmogorov’s dissipative eddies, the smallest eddies where viscous effects dominate over inertia. The size of these eddies is also controlled, though indirectly, by the shear velocity, i.e., by bed slope and water depth: with their increase the dissipative eddies shrink, similar to the viscous sublayer at the bed. Although the specific physical mechanisms securing a higher rate of nutrient delivery to the bed-attached and free-floating microorganisms are somewhat different, they also share some deep similarities. A major similarity is the potential occurrence of three uptake regimes such as *viscous-diffusive*, *viscous-convective*, and *inertial-convective*.

To test the proposed here conceptual ideas and further develop them, the laboratory experiments and high-fidelity computer simulations of interacting fields of flow velocity, substance concentrations, and substance-absorbing and substance-releasing microorganisms at relevant ranges of the Peclet and Reynolds numbers are required. The green algae could be used as a target microorganism to test the proposed concept and scaling relations, capitalising on their studies to date^4,6,7,15,24,25,29,31^. As an example, Short et al.’s^6^ experiments and numerical simulations for Volvocales in quiescent water environment could underpin laboratory tests at sufficiently high Reynolds and Peclet numbers of the flow. Similarly, insightful experiments of Shekhar et al.’s^8^ with Ciliate Protists could stimulate follow-up experiments to also cover turbulent flows. An initial step in these tests could be correlating the measured uptake rates with shear velocities and organism dimensions, identifying the presence of scaling ranges and quantifying scaling exponents. These research efforts would help clarify several issues not covered in this article, although potentially critically important for building a coherent understanding and theory. Among them we particularly would like to mention the following.

- Uncovering the interplay of mass-transfer and physical interactions between flow and organisms at the micro-scales is needed, as both these processes are likely to bear the equal importance for both bed-attached and free-floating organisms. E.g., our scaling considerations predict that increase in energy dissipation in turbulent water flows leads to increase in the uptake rate and thus organism growth. However, excessive increase in the energy dissipation is accompanied with increase in instantaneous velocity gradients that may be physically destructive for the microorganisms.
- Identification and quantitative assessment of the mechanisms contributing to the microorganisms’ dislodgement, free travel, clustering, and return to the bed are required for a more comprehensive picture.
- The estimates of various parameters (e.g., shear velocity and dimensions of specific flow sublayers, regions and scales) given in this article are indicative only and are largely based on the mean values and somewhat simplified phenomenology. In real-life situations, their actual values are likely to be highly heterogeneous in space and time, from the scales of turbulence intermittency^26,27^ to periods of flooding events. Resulting deviations from the mean values for local shear velocity, thickness of the viscous sublayer, Kolmogorov’s and Batchelor’s micro-scales may reach 200% and more, and thus this variability should be quantified and accounted for in numerical simulations and predictions. Equally important, the physical time scales need to be compared with biological time scales to identify efficiency of the proposed uptake regimes in the evolutionary transition.
- The effects of bed roughness and porosity need to be clarified and quantified as they can be important factors triggering the microorganism evolution.
- The range of water temperature in the Neoproterozoic era, from ~ 0 °C to ~ 40 °C, could result in a threefold range of water viscosity, leading to corresponding variations in the thickness of viscous sublayer at the bed and dimensions of Kolmogorov’s dissipative eddies in the outer flow. The water temperature variations could visibly modulate the delivery of nutrients towards both bed-attached and free-floating organisms, as follows from the proposed concept, e.g., from scaling relations (1) to (6).
- The proposed scaling relations in Eq. (1) to (6) for bed-attached and free-floating microorganisms suggest that there may be three ranges of the allometric relation $\:R\propto\:{M}^{\gamma\:}$ linking the metabolic rate *R* and microorganism mass *M*. This allometric relation is known as Kleiber’s Law with assumed $\:\gamma\:$ =3/4^28^. Our considerations point out the potential deviations from ¾ exponent, different in the viscous-diffusive, viscous-convective, and inertial-convective uptake regimes, being consistent with the currently available estimates^1,2^. Exploration of this matter may help in clarification of the nature of Kleiber’s Law for the microorganism range of scales.

If biological, chemical, and geological aspects of the transition to the multicellularity have been extensively studied in recent years^5^, the role of hydrodynamics and particularly turbulence at relevant time and spatial scales remains largely uncovered. A few exceptions include flow-unicell interactions in quiescent water at low Reynolds numbers^6–8^ and segregation of unicellular organisms at sub-Kolmogorov scales by Lagrangian coherent structures^32^. These evolution-oriented studies should be supplemented with several other research efforts focused on the modern flow-sediment-microorganism interactions^9,11,12,14,15,24,25^ that may appear critical in advancing present understanding of the evolutionary transition from single-cell to multi-cell organisms. The current literature suggests that there were multiple factors influencing the emergence of the multicellularity, highlighting an urgent need for a multidisciplinary and interdisciplinary studies combining and integrating biological, chemical, geological and physical understandings. This approach is aligned with Wood et al.’s^5^ vision and will undoubtedly require strengthening existing and forming new interdisciplinary teams of researchers to overcome discipline-biased interpretations. By highlighting an unconventional hydrodynamic aspect of this problem, the authors hope that this article will help to explore this approach further.

## Data Availability

The paper utilises published materials that are fully referenced in the text.
